# Constraining maximum event magnitude during injection-triggered seismicity

**DOI:** 10.1038/s41467-020-20700-4

**Published:** 2021-03-09

**Authors:** Ziyan Li, Derek Elsworth, Chaoyi Wang, EGS-Collab, L. Boyd, Z. Frone, E. Metcalfe, A. Nieto, S. Porse, W. Vandermeer, R. Podgorney, H. Huang, T. McLing, G. Neupane, A. Chakravarty, P. J. Cook, P. F. Dobson, C. A. Doughty, Y. Guglielmi, C. Hopp, M. Hu, R. S. Jayne, S. E. Johnson, K. Kim, T. Kneafsey, S. Nakagawa, G. Newman, P. Petrov, J. C. Primo, M. Robertson, V. Rodriguez-Tribaldos, J. Rutqvist, M. Schoenball, E. L. Sonnenthal, F. A. Soom, S. Sprinkle, C. Ulrich, C. A. Valladao, T. Wood, Y. Q. Zhang, Q. Zhou, L. Huang, Y. Chen, T. Chen, B. Chi, Z. Feng, L. P. Frash, K. Gao, E. Jafarov, S. Karra, N. Makedonska, D. Li, J. Li, R. Pawar, N. Welch, P. Fu, R. J. Mellors, C. E. Morency, J. P. Morris, C. S. Sherman, M. M. Smith, D. Templeton, J. L. Wagoner, J. White, H. Wu, J. Moore, S. Brown, D. Crandall, P. Mackey, T. Paronish, S. Workman, B. Johnston, K. Beckers, J. Weers, Y. Polsky, M. Maceira, C. P. Chai, A. Bonneville, J. A. Burghardt, J. Horner, T. C. Johnson, H. Knox, J. Knox, B. Q. Roberts, P. Sprinkle, C. E. Strickland, J. N. Thomle, V. R. Vermeul, M. D. White, D. Blankenship, M. Ingraham, T. Myers, J. Pope, P. Schwering, A. Foris, D. K. King, J. Feldman, M. Lee, J. Su, T. Baumgartner, J. Heise, M. Horn, B. Pietzyk, D. Rynders, G. Vandine, D. Vardiman, T. Doe, J. McLennan, Y. S. Wu, J. Miskimins, P. Winterfeld, K. Kutun, M. D. Zoback, A. Singh, R. N. Horne, K. Li, A. Hawkins, Y. Zhang, E. Mattson, D. Elsworth, K. J. Im, Z. Li, C. J. Marone, E. C. Yildirim, J. Ajo-Franklin, A. Ghassemi, D. Kumar, V. Sesetty, A. Vachaparampil, H. F. Wang, H. Sone, K. Condon, B. Haimson, W. Roggenthen, C. Medler, N. Uzunlar, C. Reimers, M. W. McClure

**Affiliations:** 1 Department of Energy and Mineral Engineering, The Pennsylvania State University, University Park, PA USA; 2 G3 Center and EMS Energy Institute, The Pennsylvania State University, University Park, PA USA; 3 Department of Geosciences, The Pennsylvania State University, University Park, PA USA; 4 Present Address: Department of Geoscience, University of Calgary, Calgary, Alberta, T2N 1N4 Canada; 5 Department of Energy, Washington, DC USA; 6 Idaho National Laboratory, Idaho Falls, ID USA; 7 Lawrence Berkeley National Laboratory, Berkeley, CA USA; 8 Los Alamos National Laboratory, Los Alamos, NM USA; 9 Lawrence Livermore National Laboratory, Livermore, CA USA; 10 National Energy Technology Laboratory, Morgantown, WV USA; 11 National Renewable Energy Laboratory, Golden, CO USA; 12 Oak Ridge National Laboratory, Oak Ridge, TN USA; 13 Pacific Northwest National Laboratory, Richland, WA USA; 14 Sandia National Laboratory, Albuquerque, NM USA; 15 Sanford Underground Research Facility, Lead, SD USA; 16 TDoeGeo Rock Fracture Consulting and Golder Associates, Redmond, WA USA; 17 University of Utah, Salt Lake City, UT USA; 18 Colorado School of Mines, Golden, CO USA; 19 Stanford University, Stanford, CA USA; 20 Mattson Hydrology LLC, Idaho Falls, ID USA; 21 Pennsylvania State University, University Park, PA USA; 22 Rice University, Houston, TX USA; 23 University of Oklahoma, Norman, OK USA; 24 University of Wisconsin, Madison, WI USA; 25 South Dakota School of Mines and Technology, Rapid City, SD USA; 26 ResFrac, Palo Alto, CA USA

**Keywords:** Natural hazards, Geophysics

## Abstract

Understanding mechanisms controlling fluid injection-triggered seismicity is key in defining strategies to ameliorate it. Recent triggered events (e.g. Pohang, Mw 5.5) have exceeded predictions of average energy release by a factor of >1000x, necessitating robust methodologies to both define critical antecedent conditions and to thereby constrain anticipated event size. We define maximum event magnitudes resulting from triggering as a function of pre-existing critical stresses and fluid injection volume. Fluid injection experiments on prestressed laboratory faults confirm these estimates of triggered moment magnitudes for varied boundary conditions and injection rates. In addition, observed ratios of shear slip to dilation rates on individual faults signal triggering and may serve as a measurable proxy for impending rupture. This new framework provides a robust method of constraining maximum event size for preloaded faults and unifies prior laboratory and field observations that span sixteen decades in injection volume and four decades in length scale.

## Abstract (web summary)

Recently triggered seismic events such as the Pohang earthquake have exceeded predictions of average energy releases by a factor of 1000. A new framework is proposed to define maximum event magnitudes as a function of pre-existing critical stresses and fluid injection volume.

## Introduction

Earthquakes occur naturally on subsurface faults in the earth’s crust, where accumulated tectonic shear stress exceeds fault strength. Natural earthquakes are caused by the slow buildup of tectonic stresses over geological time but may additionally be triggered by a relatively small stress perturbation if the affected region is critically stressed^1,2^. The frequency of induced earthquakes has increased dramatically over the past few years due to industrial-scale injection of fluids such as in hydraulic fracturing, enhanced geothermal systems, and wastewater disposal^3–9^ where small perturbations have prematurely triggered earthquakes. Known mechanisms for such earthquakes include: (1) elevated pore pressures that reduce effective normal stress, thus decreasing the fault strength; and (2) local encapsulated stress halos that exceed far-field stresses, causing shear failure without changing the fault strength^3^. Moreover, the maximum magnitudes of fluid injection associated earthquakes have been substantial, such as the 2011 Mw 5.7 Prague earthquake^6^, the 2016 Mw 5.1 Fairview earthquake^10^, the Mw 5.0 Cushing earthquake^11^, the Mw 5.8 Pawnee earthquake^12,13^, and the 2017 Mw 5.5 Pohang earthquake^14,15^.

Methods to forecast the largest anticipated magnitude of injection-induced earthquakes have been proposed by previous studies based on the total injected volume^16,17^, inferred dimensions of the stimulated volume^18,19^, theoretical scaling relations^20^, and changes in seismic-activity rates linked by probabilistic approaches^21^. The greatest advantage in scaling magnitude with total injected volume is that it can be applied prior to injection, without a priori knowledge of either the fault**/**reservoir dimension or history of seismicity. This classic theory^16,17^ proposes that the maximum seismic moment (*M*_0_^max^) cannot exceed an upper bound defined by the product of the total injected fluid volume (∆*V*) and the shear modulus (*G*) of the affected zone, i.e., *M*_0_^max^ = *G*∆*V*. However, recent observed injection-induced earthquakes associated with enhanced geothermal systems^22,23^, hydraulic fracturing^24^, and a purposely reactivated fault in a field pilot experiment^4^ exhibit magnitudes significantly larger than this threshold. The mechanisms causing this underestimation are still not well understood.

The following redefines the upper limit for moment magnitudes of fluid-injection-induced earthquakes and reveals the underlying mechanisms. Here we design and conduct laboratory experiments reproducing fluid pressurization induced slip on Precambrian schists (EGS-Collab)^25^. We explore constraints on the upper threshold of moment magnitudes through a rigorously structured experimental suite, in which we correlate moment magnitudes with initial stress state, total injection volume, and slip versus aperture opening. The experiments generate unexpectedly large seismic events for modest fluid pressurization and our analyses define constraints for properly estimating the maximal probable seismic moment magnitude.

## Results and discussion

### Experimental protocol

We reactivate prestressed laboratory faults by fluid pressurization as an analog for fluid-injection-induced earthquakes using a double-direct-shear apparatus enclosed within a pressurized core holder^26^ (Supplementary Fig. 1). Injected fluid volume controls over-pressurization and the resulting shear displacement provides a proxy for deformation moment magnitude. The experiments are conducted under two end-member boundary conditions relevant to induced seismicity^3^ (Supplementary Fig. 2): constant shear stress (CSS) that is broadly representative of normal faulting under invariant overburden stress, and zero displacements (ZD) boundary conditions to characterize reverse faulting under applied horizontal deformation. Strike-slip faulting would be close to conditions of ZD (rate) control but these boundary conditions do not influence the initiation nor progress to failure of any of these faulting geometries. ZD refer to the piston/load-point, allowing the fault to displace as it dissipates the elastic strain energy of the system. Similarly, the natural fault analog is ZD in the far-field, infinitely far from the displacing fault. Samples are confined at a constant normal stress of 3 MPa before performing the following stages: Stage I: Shear loading is initially applied at a constant rate of 10 µm/s to shear-mobilize the fractures to post-peak steady-state; Stage II: Shear stress is then lowered to a set proportion of the peak strength; Stage III: A series of fluid-pressurization pulses are applied to induce shear slips. Detailed information for each test is shown in Table 1 and typical evolutions of shear stress, pore pressure, and shear displacement are shown in the supplementary materials.

**Table 1:** Experimental results.

Exp. No.	Ʈp (MPa)	Ʈss (MPa)	Ʈ0 (MPa)	%	µp	µss	Initiate Pp (MPa)	Initial aperture (µm)	Normal stiffness (kN/µm)
CSS1	1.49	1.25	0.91	0.61	0.50	0.42	0.8	30.397	0.092
CSS2	1.58	1.52	1.27	0.80	0.53	0.51	0.5	29.356	0.080
ZD1	1.62	1.52	1.41	0.87	0.54	0.51	0.2	25.248	0.092
ZD2	1.49	1.40	1.34	0.90	0.50	0.47	0.2	29.339	0.086

### Experimental results

Hydraulic aperture of the laboratory fault is estimated from the cubic law^27^, adjusted for the double fracture configuration unique to our system^26^. Typical initial aperture values range from 25 to 30 µm. During the initial shear-mobilization stage, we observe that frictional strength from all experiments exhibits a peak frictional strength (ratio of shear stress to confining stress) from 0.50 to 0.54 that returns to a residual steady-state value of ∼0.42–0.51 (Supplementary Fig. 2). Fracture normal stiffness (*K*_N_) is measured in the range 0.080~0.092 kN/µm (Supplementary Fig. 3). The shear stiffness of the experimental system (*K*_s_) is 0.067 kN/µm^28^. The physical properties measured for each experiment are listed in detail in Table 1.

### Fluid injection-induced slip

**Fig. 1:**
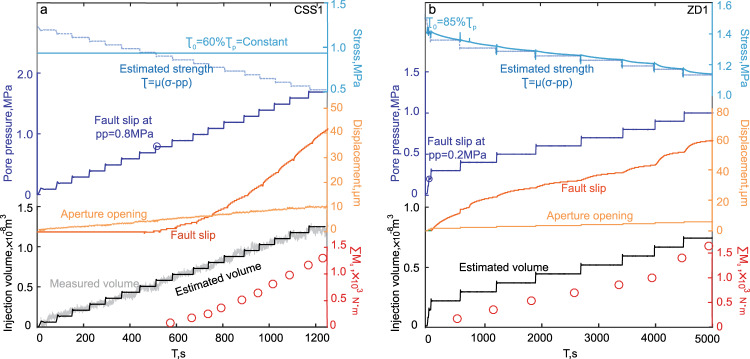
Typical observed fault displacement and cumulative moment over time as induced by fluid injection. The reactivated laboratory faults are under boundary conditions of (**a**) constant shear stress and (**b**) zero displacement. Measured variables and axes are color-coded to show the time evolution of fault fluid pressure, injection volume, fault slip and dilation, instantaneous strength, and cumulative moment magnitude.

The typical evolution of shear stress, slip displacement, pore pressure, injection volume, and cumulative moment magnitudes during Stage III for both boundary conditions are summarized in Fig. 1. For the experiment under CSS boundary conditions (CSS1, Fig. 1a), the peak strength is measured at τ_*p*_ = 1.49 MPa in Stage I. The stress is then decreased to ≈60%τ_*p*_ in Stage II and kept constant for the entire Stage III. De-aired water is sequentially injected into the fully saturated fault at 0.1 MPa/min (dark blue curve, Fig. 1) sufficiently slowly to allow a uniform pressure distribution. During fluid pressurization, shear strength decreases with reduced effective normal stress (dashed blue curve, Fig. 1). Slip is induced when the applied shear stress exceeds shear strength. The first fluid-induced slip occurs at ~500 s into the test, when pore pressure is elevated to 0.8MPa. As the fault is further weakened by increasing pore pressure, shear displacements in the following pressurization steps are increased, shown by the ascending slope (orange, marked as fault slip, Fig. 1a). Fluid pressurization also promotes aperture opening or normal dilation (yellow, marked as aperture opening, Fig. 1a). The cumulative shear displacement exceeds normal displacement at ~750 s, serving as an important threshold indicator which will be discussed later. The total injected volume (gray curve, Fig. 1a) in the fracture is evaluated from the slip area and measured aperture opening (Eq. (3)). This same volume (black curve, Fig. 1a) is also evaluated using pore pressure and the normal fracture stiffness (Eq. (4)). The agreement between the black and gray curves shows the congruence between these two independent methods of estimating injected volume. Cumulative deformation moment magnitudes (red) for each slip are calculated as the sum of the moment magnitudes from all previous slips (Eq. (5)).

Typical evolutions under the zero-displacement boundary condition (ZD1) are shown in Fig. 1b. Shear loading is halted before fluid pressurization, representing shear stress relaxation during induced slips^29^. The shear strength is applied at 1.62 MPa in Stage I and unloaded to ~85%τ_*p*_ in Stage II. In Stage III, fluid pressure is elevated in 0.1 MPa increments until slip is induced. The fault is allowed to slip then self-arrest before the next pressurization increment is applied, during which shear stress relaxes and gradually drops to/below the corresponding frictional fault strength defined by effective stress. The fault reactivates almost immediately after fluid pressurization (~0.2 MPa) since the prevailing shear stress approaches the critical stress, i.e., peak strength. Fault slip is rapid, immediately after reactivation, and slows towards the end of each pressurization step. This contrasts with the continuous acceleration of fault sliding under the CSS boundary condition, where driving stress remains. Fault shear displacement (slip—orange) exceeds dilation (aperture opening—yellow) early in the loading cycle and continues to grow at a faster rate. The cumulative injection volume is recovered from effective stress and fracture stiffness (see calibration against measured volume CSS in Fig. 1a) as shown as the black curve in Fig. 1b.

### Secondary slip during pressurization

**Fig. 2:**
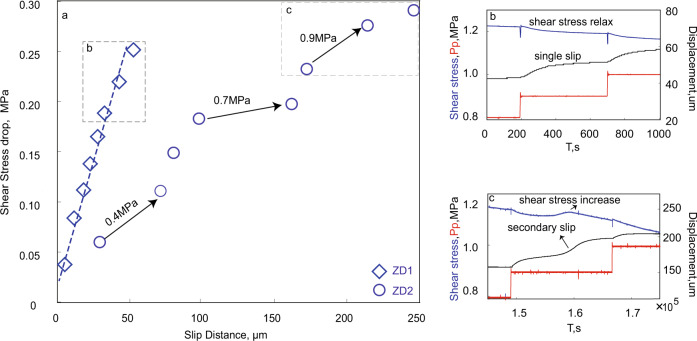
Shear stress drop/relaxation during fluid pressurization. **a** Shear stress drop vs. slip displacement for tests ZD1 and ZD2. Experiment ZD1 shows a constant slope during fluid pressurization, while ZD2 features slope changes for the 0.4, 0.7, and 0.9 MPa pressurization steps. Pressurization step at 0.9 MPa for ZD1 (**b**) and ZD2 (**c**) are shown for comparison.

We show shear stress drop/relaxation as a function of displacement under the ZD boundary condition in Fig. 2. Data from experiment ZD1 (initial stress at 85% τ_*p*_, blue diamonds in Fig. 2a) show that the shear stress drop scales near-linearly with slip distance. However, for ZD2 (initial stress at 90% τ_*p*_, blue circles in Fig. 2a) the relationship features some changes in slope at a few pressurization steps (the corresponding pore pressure values are annotated). To reconcile the possible mechanism, the stress-displacement relationship during the 0.9 MPa pressurization step for ZD1 and ZD2 are shown in Fig. 2b and c, respectively. Notable in ZD2 is that shear stress initially relaxes but heals followed by a secondary stress drop, resulting in a larger total displacement. A plausible mechanism may be due to the presence of local asperities resisting slip during the pressurization step and building-up shear stress within that pressurization step. The stored energy is released when the resisting asperities fail, resulting in an increased total shear displacement during that step.

### Unexpectedly large excess moment magnitudes

**Fig. 3:**
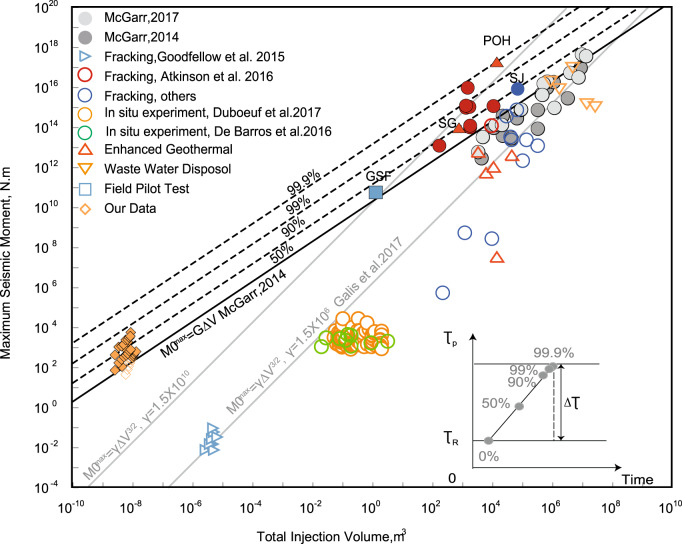
Maximum seismic moment versus total injection volume for fluid-injection-induced earthquakes. Black line defines the upper limit of the seismic moment for assumed average stress. The black dashed lines represent solution with *c* values as shown in the lower-right inset. Gray solid lines represent maximum seismic moment and are added for completeness with two different γ values.

Initial stress state of the fault is one of the key parameters controlling the magnitude of the induced earthquake. However, the classic upper limit of maximal moment magnitude^16,17^ simply assumes the initial shear stress as the average of peak strength and residual stress—midway in the window defining the stress drop in the natural earthquake cycle (50% point in Fig. 3a inset). Thus, a driving stress change of half the total potential stress drop is required to drive the fault to failure and to then deliver the full stress drop as a potentially seismic event. However, this ignores the possibility of recovering this same “full” stress drop from a fault that is already “primed” close to failure before this final triggering occurs (e.g., Fig. 3 inset at 99.9%). Considering all possible initial stress states within this window, we denote a stress ratio (**c**) as the proportion of the full stress drop magnitude. Therefore, an increase in pore pressure given by Δ*P* is sufficient to cause fault failure and rupture as μΔ*P* = (1 − *c*)Δτ, where μΔ*P* is the shear strength reduction due to the increased pore pressure; (1 − *c*)Δτ is the stress difference between the initial shear stress and the shear strength. If we substitute this equation for $\Delta {\mathrm{P}} = \frac{{\Delta {\uptau}}}{{2{\mathrm{\mu }}}}$ (Eq. (3)^17^), then the maximum anticipated moment magnitude is redefined as ${\mathrm{M}}_0^{{\mathrm{max}}} = \frac{1}{{2(1\, -\, {c})}}{\mathrm{G}}\Delta {\mathrm{V}}$ representing an increase in the anticipated moment magnitude for triggering (*c* > 50%) relative to that for an induced event (*c* = 50%). This relation accommodates a spectrum of events larger than *M*_0_^max^ = *G*Δ*V* by relaxing the requirement that the initial stress is mid-way within the stress-drop window (Fig. 3, inset)—but all other constraints apply^16,17^. It assumes that failure occurs on the most critically oriented plane relative to the stress field, is limited to a single rupture event that is fully contained within the uniformly pressurized region and described by Byerlee’s law (μ ~ 0.6 and no cohesion) and with no distinction made between stable (aseismic) or unstable (seismic) rupture. Nonetheless, despite these constraints, the relations (both original^16,17^ and this revised form) show a remarkable congruence with observations.

We assign a scale factor, $\lambda = {\mathrm{\mu }}\frac{{K_N}}{{K_s}}$, to the experimental measured seismic moments to account for the differences in geometry and constraints between laboratory faults and natural faults. A detailed derivation of this is presented in the supplementary note. The normalized maximum seismic moment for our laboratory slip events of, *M*′_0_^max^ = *M*_0_^max^/*λ*, are shown in the context of a variety of injection-induced earthquakes, as a function of injected volume, in Fig. 3. The upper limit for the seismic moment for an induced event^17^ is shown as the black solid line (*c* = 50%). Indeed, the majority of reported fluid injection-induced earthquakes (hollow and gray markers, Fig. 3) are constrained by the predicted threshold. Events with moment magnitudes exceeding this threshold, include the Fort St. John earthquake in BC, Canada^30^ (SJ), the St. Gallen earthquake in Switzerland^22^ (SG), a scientific fluid injection field experiment in southeast France^4^ (GSF), and the Pohang earthquake in South Korea^23^ (POH). These triggered events may be attributed to a higher initial shear stress than assumed in the mean-stress analysis. To illustrate this argument, we plot *c* = 75%, 99%, and 99.9% (successively critically over-stressed conditions) as the dashed gray lines in Fig. 3. The threshold maximum moment magnitude correspondingly increases for a given volume of injected fluid as c increases—with all observed events constrained by this extended upper limit. The maximum deviation from the mean-stress norm is the Pohang earthquake—apparently triggered when fluid was injected into a near-critically stressed subsurface fault zone^23^. The injection of water (~10^4 ^m^3^) at shallow depth (4~5 km) triggered an M_w_5.5 mainshock with an estimated seismic moment of ~1.7 × 10^17^ N m. However, the mean-stress threshold (*M*_0_^max^ = *G*Δ*V*) incorrectly estimates the maximum event magnitude to be limited to *M*_0_ ~ 3 × 10^14^ N m—approximately three-orders-of-magnitude lower than actuality. The observed seismic moment (~1.7 × 10^17^ N m) corresponds to the stress being within 0.1% of the strength of the fault (*c* = 99.9%), as noted in the prior analysis. This is consistent with the conceptualization that a relatively small volume of injected fluid can trigger slip on a fault in a near-critical state of stress state, that then recovers the full stress drop of failing fault.

**Fig. 4:**
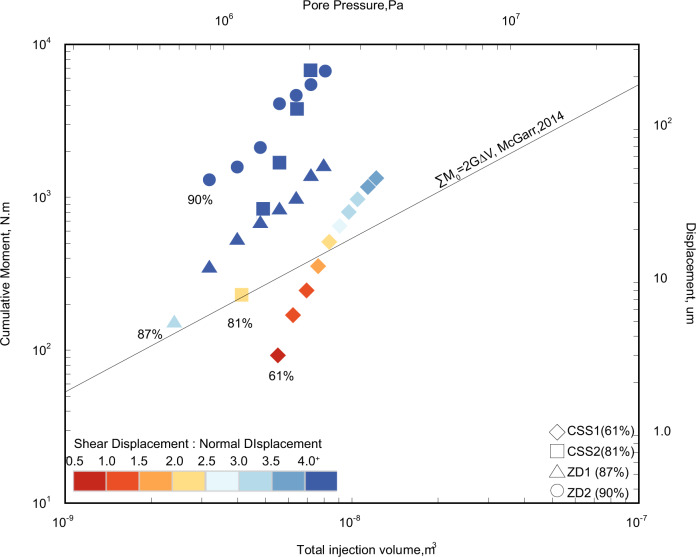
Cumulative moment magnitude versus total injection volume for all fluid pressurization steps in our experiments. Symbol colors from hot to cold represent an increasing ratio of slip displacement to fault dilation. Specifically, in CSS1, the cumulative moment magnitude exceeds the classic limit when this ratio is >2. Initial stress ratios τ/τp are shown as percentages.

There are two conditions that may contribute to the occurrence of these observed unexpectedly large moment magnitude events: the form of the stress boundary conditions and local asperities. We adopt CSS and ZD boundary conditions to represent constant overburden stress (normal fault) or stress relaxation (reverse fault). Generally, we can expect a larger moment magnitude from the induced events if constant stress is maintained (i.e., the CSS boundary condition). This is because, the increased pore pressure reduces fault strength and therefore increases the strength deficit relative to the CSS—driving rupture of the fault and its acceleration. Conversely, for the ZD boundary condition, the shear stress will relax immediately upon fault reactivation. This stress regime allows the fault to creep and potentially self-arrest. These two fault displacement modes are illustrated by the different slopes in the cumulative moment magnitude versus total injected volume relations of Fig. 4.

Secondary slips within a single fluid pressurization step are observed only under ZD boundary conditions (Fig. 2c). We suspect that this behavior is promoted by the resistance of local asperities. During fault creep under the ZD boundary condition, local asperities will accumulate shear stress and generate secondary slip when the increased local shear stress exceeds fault strength. Such secondary slips utilize the strain energy stored in the asperity and contribute to a longer slip distance before the fault self-arrests—thus resulting in larger cumulative deformation and moment magnitudes.

### Exceeding the classic threshold as fault slip outpaces dilation

We have already noted that fluid pressurization results in dilation of the fault (tensile failure and hydraulic-jacking) followed by slip (shear failure and hydro-shearing). The cumulative moment may be calculated as ${\sum} {{\mathrm{M}}_0 = \frac{1}{{(1\, -\, {c})}}{\mathrm{G}}\Delta {\mathrm{V}}}$, and for mean-stress prior to failure *c* = 50% and results in ${\sum} {{\mathrm{M}}_0 = 2{\mathrm{G}}\Delta {\mathrm{V}}}$, equivalent to the classic threshold expression. In our experiments, the total injected fluid volume is primarily accommodated by fault dilation, defined as Δ*V* = *aA* where *a* is the change in fault apertures (two faults in our testing configuration) and *A* is the area of the slipping patch. The total moment magnitude resulting from the reactivation of a patch of area *A* is ${\sum} {{\mathrm{M}}_0 = {\mathrm{AGu}}}$^29^, where *u* is the shear slip displacement. Equating this to ${\sum} {{\mathrm{M}}_0 = 2{\mathrm{G}}\Delta {\mathrm{V}}}$ yields ${\mathrm{u}} = 2a$. This relationship implies that for faults under initial conditions of average stress, the cumulative moment magnitude can exceed the classic predicted maximum (${\sum} {{\mathrm{M}}_0 = 2{\mathrm{G}}\Delta {\mathrm{V}}}$) as the slip displacement (*u*) equals or exceeds twice of the fault dilation (2a). We observe this behavior in experiment CSS1 (Fig. 4). The calibrated maximum moment magnitude grows with the ratio of slip over aperture opening. The cumulative moment magnitude of experiment CSS1 (60% τ_p_) exceeds the classic moment threshold (${\sum} {{\mathrm{M}}_0 = 2{\mathrm{G}}\Delta {\mathrm{V}}}$) when the ratio of slip to aperture opening exceeds 2. For all other experiments the slip and aperture opening ratios are all >2 from the initiation of the fluid injection. This is plausibly a result of the initial stress states for these experiments approaching the corresponding critical stress state. This finding emphasizes the importance of knowing the initial stress state to define the likely rupture magnitudes—but also presents a role for monitoring stress and deformation in fluid pressurization zones as a metric to index anticipated rupture energetics. Unexpectedly large events may result as the magnitude of fault slip displacement exceeds double the fault dilation—as apparent in our experiments.

### Bridging the scale-gap

Laboratory faults are clear idealizations of the complexity in both form and response of natural faults. Laboratory experiments apply an unusual constraint on applied and measured stresses, fluid pressures and displacements albeit at reduced scale and complexity. Fluid pressures may be non-uniform^31^ or uniform within the sample—the latter aiding the direct recovery of the observed rheology and simplifying the evaluation of constitutive response, as in this study. The pressurized area is always confined within the laboratory fault even when the entire fault slips. This can be considered as the critical condition of run-away rupture under laboratory conditions, whereas the rupture area can extend beyond the pressurized zone in natural faults if a critical pressure is reached, and possibly transit to runaway rupture that results in a larger seismic event^20,32^. In addition, a broad array of important physical characteristics such as interseismic strengthening^33^, healing^34^ and sealing^35^ are readily represented in laboratory experiments as analogs of behavior in situ. However, the reduced scale and idealized loading geometry of laboratory faults mean that the roles of branching, asperities, fault nucleation and propagation together with fault-fault interactions in natural faults are more challenging to accommodate— except in microcosm. A key necessity is in accommodating the intrinsic length scale of the rupture of natural faults, as this impacts the effective geometric stiffness of the fault and controls the style of failure—aseismic through seismic. Although geometrically dissimilar, the rigid body deformations of a thoroughgoing laboratory fault and the heterogeneous deformation around a penny-shaped-crack of finite extent, representing a natural fault, may be equated by matching system stiffnesses. Matching stiffness contrasts between fault element and the geometric stiffness of the fault/loading-system allows the full spectrum of fault slip modes to be replicated in the laboratory—from stable slip to slow and fast earthquakes^36^.

### Pressurization constrained versus unconstrained fault rupture

The McGarr model^16,17^ and our modification for critical stressing equate the elastic strain energy recovered from the overpressured volume where frictional-only failure is constrained within that volume of the solid. The fault cannot propagate beyond the region of fluid pressurization and the magnitude-volume scaling relation is as *M* ∝ Δ*V*^1^. More complex physics-based models^20^ accommodate fault propagation beyond a region of non-uniform fluid pressurization where the shear failure again propagates in a region of tectonic prestress but against a mode II/III fracture toughness at the rim of the propagating fault. Such models are capable of accommodating contained, arrested and runaway ruptures with mechanism-based distillations of such models^20^ parameterizing the scaling relation as *M* ∝ Δ*V*^3/2^. We include broad limits of this model^20^ for comparison in Fig. 3. The assumptions of these two models^16,20^ are intrinsically different but both are broadly consistent with current observations of *M*−vs−Δ*V* relationships within the range 2 < *M* < 6. It is potentially important to discriminate between these two scaling relations as *M*−Δ*V* predictions diverge for events outside this range of congruence. This is especially true at the upper limit of injection volumes and event magnitudes (*M* > 4) where projections of the seismic moment are larger when faults propagate beyond the pressurized volume^20^ relative to those that are constrained to within it (this paper). However, field data remain equivocal within the range of full-scale observations (2 < *M* < 6), are absent at larger magnitudes (*M* > 6), and with only small scale in situ experiments^4,37,38^ and laboratory observations^39^, this paper as scaling discriminants. Of these, the laboratory observations potentially discriminate the most definitively between the Δ*V*^1^ and Δ*V*^3/2^moment-volume scaling relations (Fig. 3). These two scaling models are for rupture either contained^16,17^ or uncontained^20^ within the fluid pressurized volume. The “contained” model is for uniform fluid pressurization, a uniform pre-stress, spatially uniform shear slip on the fracture and with neither cohesion nor mode II fracture toughness—conditions exactly matched by our experimental configuration. The “uncontained” model represents a planar fault propagating within a uniform prestress from a central highly localized region of non-uniform fluid pressure against both frictional resistance and mode II fracture toughness at the rim of the propagating fracture. This model^20^ also identically represents the conditions of the matched experimental data^39^ here representing mode I propagation of a hydraulic fracture from a localized point-source wellbore but absent a pre-existing fault and shear prestress. The remarkable fit of these two distinctly different scaling models to the respective experimental geometries and configurations hint to their application at the field scale. The “contained” model honors the configuration of broad pressurization of a weak fault, potentially representing injection into a high permeability fault with low or zero effective fracture toughness or cohesion. Conversely, the “uncontained” fault propagation model best represents localized pressurization on a strong fault, characterized by low permeability (or rapid fluid pressurization rate) and high cohesion or fracture toughness. The distinct differences between *M*−vs*−*Δ*V* response in these two experimental configurations and represented by these two models diminishes with increased injection volume and seismic moment as the different scaling models converge—before again ultimately diverging.

This convergence of the *M* ∝ Δ*V*^1^ and *M* ∝ Δ*V*^3/2^ scaling regimes at increased moment/injection-volume leaves as equivocal the principal modes of response at the field scale. However, it does suggest that the magnitude of critical tectonic shear stress within the reservoir is a key property in defining behavior in addition to merely the injection volume, frictional characteristics of the fault and stiffness of the reservoir. Of these, reservoir stiffness and fault friction are narrowly constrained for real faults and injected volume is typically known—leaving the magnitude of pre-stress as a final crucial property to determine—in addition to potential controls of permeability, alluded to earlier.

### Diagnostic signals and precursory features

This study has demonstrated that the initial stress state on the fault is a dominant factor in linking the maximum moment magnitude to injected volume. This simple analysis assumes a uniform distribution of fluid pressure and therefore discounts the influence of pumping rate in the triggering process and the mitigative impact of slowing or ceasing injection. However, these are not intrinsic limitations to the approach, as pre-existing stress must surely influence event magnitudes for non-uniform distributions of pressure. Thus, determining the pre-existing stress that “primes” the system for failure is a key parameter to determine—and in particular the magnitude of this absolute stress within the window of the interseismic loading cycle (Fig. 3). Thus, defining the stress ratio, *c*, requires that the maximum in situ stress, stress drop and peak (or minimum) interseismic stress all be determined—to uniquely locate the existing stress within the stress-drop window. The absolute magnitude of the pre-loading stress may be determined from regional indicators or from in situ stress measurements. The seismic stress drop may be recovered from regional seismicity. And peak interseismic stress may be recovered from that same seismicity or from the measurement of fault strength. Noted is that all such evaluations are fraught with their appropriate uncertainties and interdependencies on selected parameters and assumptions. In addition, we also note the tantalizing observation that the cumulative moment can exceed the predicted threshold when the ratio of slip to aperture dilation evolves to >2. However, such an indicator for exceedance of the maximum moment would be difficult to measure in a natural fault system. Finally, we also note the potential for aseismic deformation that would reduce the cumulative seismic moment and concomitantly reduce the seismic hazard.

## Methods

### Experimental apparatus

We conduct injection-induced slip experiments on samples of schist using a novel double direct shear apparatus (Supplementary Fig. 1). We configure the bulk samples into an assembly of two half cylinders sandwiching a central prism to create a double-direct-shear fracture geometry. The contacting rock interfaces represent fault analogs that are uniformly roughened with 60-grit aluminum oxide powder to produce repeatable and controlled initial roughness. The assembled core is hydraulically isolated by a latex jacket and installed in a pressure vessel. Normal stress, shear stress, and pore pressure are controlled independently by servo-controlled precision hydraulic pumps (A, B and C). Fault slip is observed though shear displacement, monitored by a linear variable differential transducer (LVDT) connected to the loading piston (driven by Pump B). Dilation of the faults (normal displacement) is recovered from circumferential strain recorded through a strain-belt—a strain gage attached to a thin (0.127 mm in thickness) aluminum shim wrapped around the assembled core that covers the two fractures^26^.

### Experimental strategy

The experiments are initialized by gradually increasing confining pressure to 3MPa via pump A, then held constant under servo-control. We allow the analog faults to fully compact by measuring aperture change through the strain-belt. The normal stiffness of the analog fault/fracture is calculated as:

1 $${\mathrm{k}}_{\mathrm{n}} = \frac{{{\mathrm{A}}\Delta {\upsigma}_{\mathrm{N}}^\prime }}{{{\mathrm{u}}_{\mathrm{n}}}} = \frac{{{\mathrm{A}}\Delta \left( {{\upsigma }}_{\mathrm{N}} - {\mathrm{p}}_{\mathrm{p}} \right)}}{{\Delta a}}$$

where fracture normal stiffness k_n_ is the rate of change in normal force with respect to variation in normal displacement (fracture closure) u_n_, given by the change in the aperture Δ_*a*_ of two fractures in the double-direct-shear sample core. A sample stiffness calculation is included in Supplementary Fig. 3. The completion of the initial compaction is marked by a leveling in the strain displacement. After establishing this initial compaction, de-aired water is injected from the upstream pump (regulated by pump C, initially at 20 KPa), flowing through the dual compacted rock interfaces (analog faults/fractures) to the downstream (atmospheric pressure) until fully saturated.

The experiments are conducted in two stages with typical procedures shown in Supplementary Fig. 2. In Stage (1), shear loading is applied at a constant rate to shear-mobilize the fractures to post-peak steady-state and to determine the peak strength (Ʈp), steady-state stress (Ʈss), and steady-state fracture aperture (b_h0_). In Stage (2), we systematically introduce injection-induced slip in the sample core to evaluate the evolution of slip behavior and moment magnitude as a function of pore pressure, injected volume, and corresponding stress drop.

Stage (1) initiates after saturating the fractures when shear stress is applied through pump B, driving shear displacement at a constant velocity of 10 µm/s. The stress on the sample increases until peak strength with the loading halted as the sample reaches a post-peak steady state. During this loading stage, hydraulic aperture (a_h_) is estimated via the cubic law^27^, accommodating the double fracture configuration unique to our system as:

2 $$a_{\mathrm{h}} = \left( {\frac{{\left( {{\mathrm{Q}} + {\mathrm{whV}}_{\mathrm{s}}} \right)}}{2}\frac{{\mathrm{l}}}{{\Delta {\mathrm{P}}}}\frac{{12{\upmu}}}{{\mathrm{w}}}} \right)^{1/3}$$

where Q is the flow rate of Pump C, w, h, and l are width, height, and length of the central prism, respectively. The term (whV_s_) represents a flow rate correction for the intrusion of the central prism into the jacketed fluid reservoir^28^ where V_s_ is the slip velocity. ΔP is the pressure difference measured along the flow path and μ is the viscosity of the fluid (water, 8.9 × 10^−4^ Pa s). The hydraulic aperture at the conclusion of Stage I is defined as the “initial” hydraulic aperture for stage (2), denoted as initial $a_{h_0}$.

In stage (2), shear stress is decreased to a prescribed primed- or critical-stress state, i.e., the ratio of current stress state to the peak strength before failure (e.g. 65–90% percent of peak strength). Shearing of the sample is halted at this pre-defined “primed-stress” prior to fluid injection to replicate the variety of initial stress conditions anticipated in nature. After stabilizing the fractures, pore pressure is elevated to induce shear slip in multiple steps. Loading is applied either as CSS or ZD with the servo control mechanism turned off.

During fluid injection, the downstream fluid outlet from the sample is closed so that the total injected volume can be calculated as

3 $$\Delta {\mathrm{V}} = {\mathrm{A}}\Delta a$$

where A is the cross-sectional area of each fracture (5.72 × 10^−4^ m^2^), and Δ*a* is the total aperture change as fluid is injected, measured by the strain-belt. Alternately, Eq. (3) may be combined with Eq. (1) to estimate the cumulative injection volume into the fractures using the fracture normal stiffness, as

4 $$\Delta {\mathrm{V}} = \frac{{{\mathrm{A}}^2\left( {\sigma _{\mathrm{N}} - {\mathrm{p}}_{\mathrm{p}}} \right)}}{{{\mathrm{k}}_{\mathrm{n}}}}$$

The slip displacement that results when pore pressure is elevated is monitored via LVDT. Under CSS loading, pore pressure is increased at a rate of 0.1 MPa/min (Supplementary Fig. 2a) until the fracture is reactivated. The reactivated fracture slides continuously due to the constant external shear stress. In contrast, under the zero-displacement condition, the shear stress is reduced to the pre-defined “primed-stress” and the constant stress servo-control is then turned off. When increasing the pore pressure, shear stress drops as the fracture reactivates and slip halts as shear stress drops below the current shear strength, as illustrated in Supplementary Fig. 2b.

For each pore pressure step, we monitor the resulting slip and related moment magnitude with cumulative moment magnitude recovered from

5 $$\sum {\mathrm{M}}_{0} = {\mathrm{GA}} \sum {\mathrm{u}}$$

where M is the seismic moment magnitude, G is the shear modulus of the sample, A is slip patch area, and u is the measured slip displacement^40^.

## Supplementary information

Supplementary Information

## Data Availability

Data are available based on reasonable request to the authors.
