# Supplementary material for: Constraining maximum event magnitude during injection-triggered seismicity
Source: Nat Commun. 2021 Mar 9;12:1528. doi: 10.1038/s41467-020-20700-4 (PMC7943564; doi:10.1038/s41467-020-20700-4)
Supplement: Supplementary file 1 — Supplementary Information [file 41467_2020_20700_MOESM1_ESM.docx]

Supplementary Information for:

Constraining maximum event magnitude during injection-triggered seismicity

Ziyan Li^1,3^*, Derek Elsworth^1-3^, Chaoyi Wang ^1,3^*, EGS-Collab^4^

^1^Department of Energy and Mineral Engineering, The Pennsylvania State University, University Park, PA, USA

^2^Department of Geosciences, The Pennsylvania State University, University Park, PA, USA

^3^G3 Center and EMS Energy Institute, The Pennsylvania State University, University Park, PA, USA

^4^EGS-Collab Team – Author list appended at the end of the manuscript.

Supplementary Figures
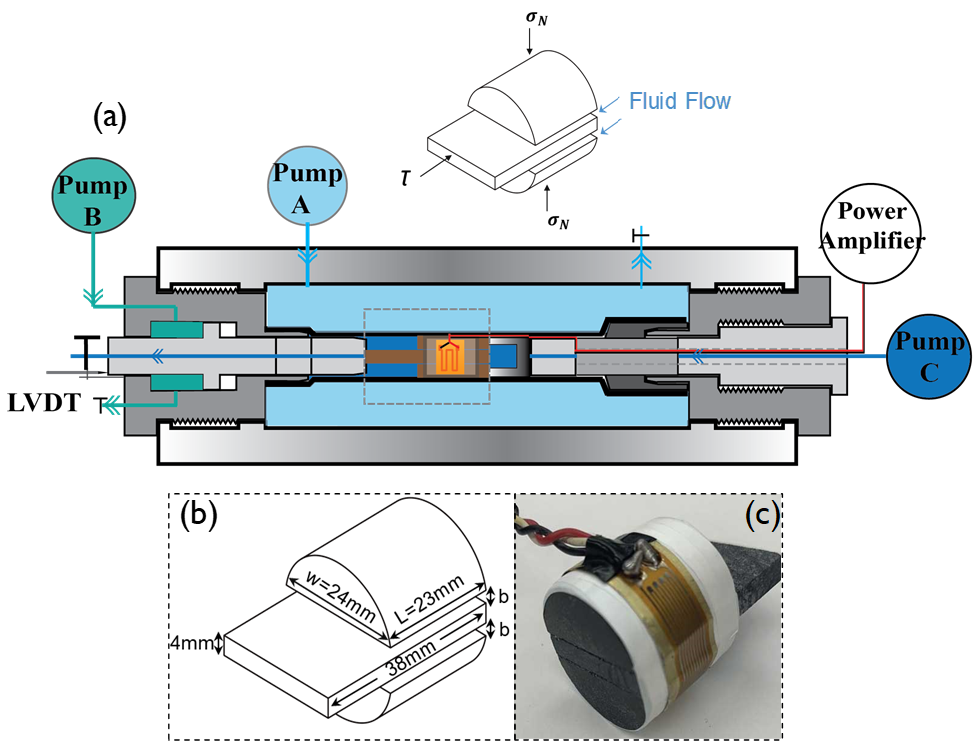


**Supplementary Figure 1-Experimental configuration.** Confining stress is applied by pump A, shear stress in applied by pump B, and upstream pore pressure is applied by pump C. Shear displacement is measured by a linear variable differential transformers (LVDT) mounted against the shear loading piston. Normal displacement is monitored by strain gauge (b) Schist sample with detailed dimensional information. (c) Sample with strain gauge assembled.


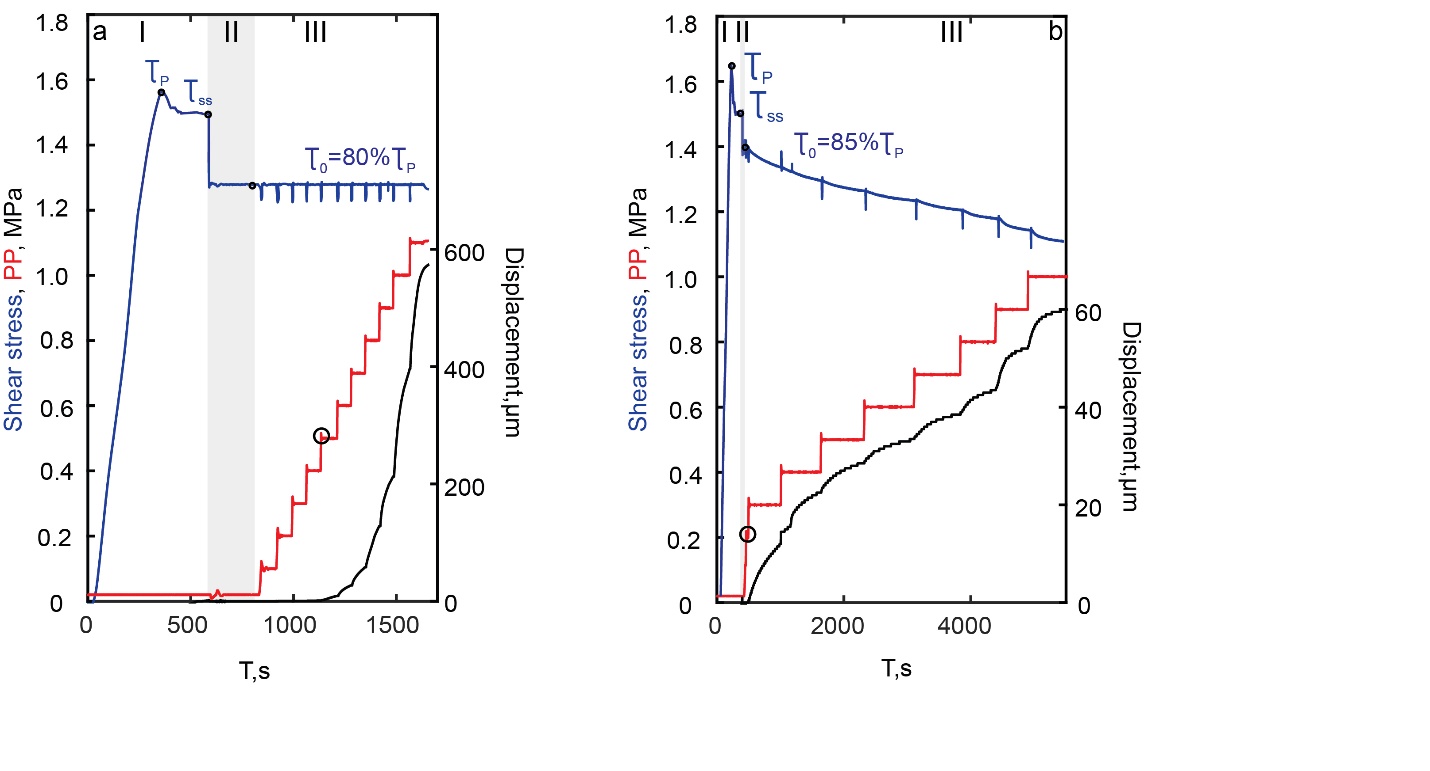


**Supplementary Figure 2 - Typical experimental curves under two boundary conditions.** Data for one complete experiment show the evolution of shear stress (blue), pore pressure (red), and slip distance (black) as a function of time under different boundary conditions: (a) constant shear stress and (b) zero displacement condition. Stage I shows that shear is initiated by a constant loading rate of 10µ/s, passing peak strength (τ_p_) until reaching steady state(τ_ss_). Stage II represents shear stress is then decreased to a primed stress (τ_0_), and last for~220s for Fig.b, and ~230s for Fig.b. ,In Stage III, fractures are reactivated by elevating pore pressure systematically. The black dot shows the pore pressure when fault is reactivated.


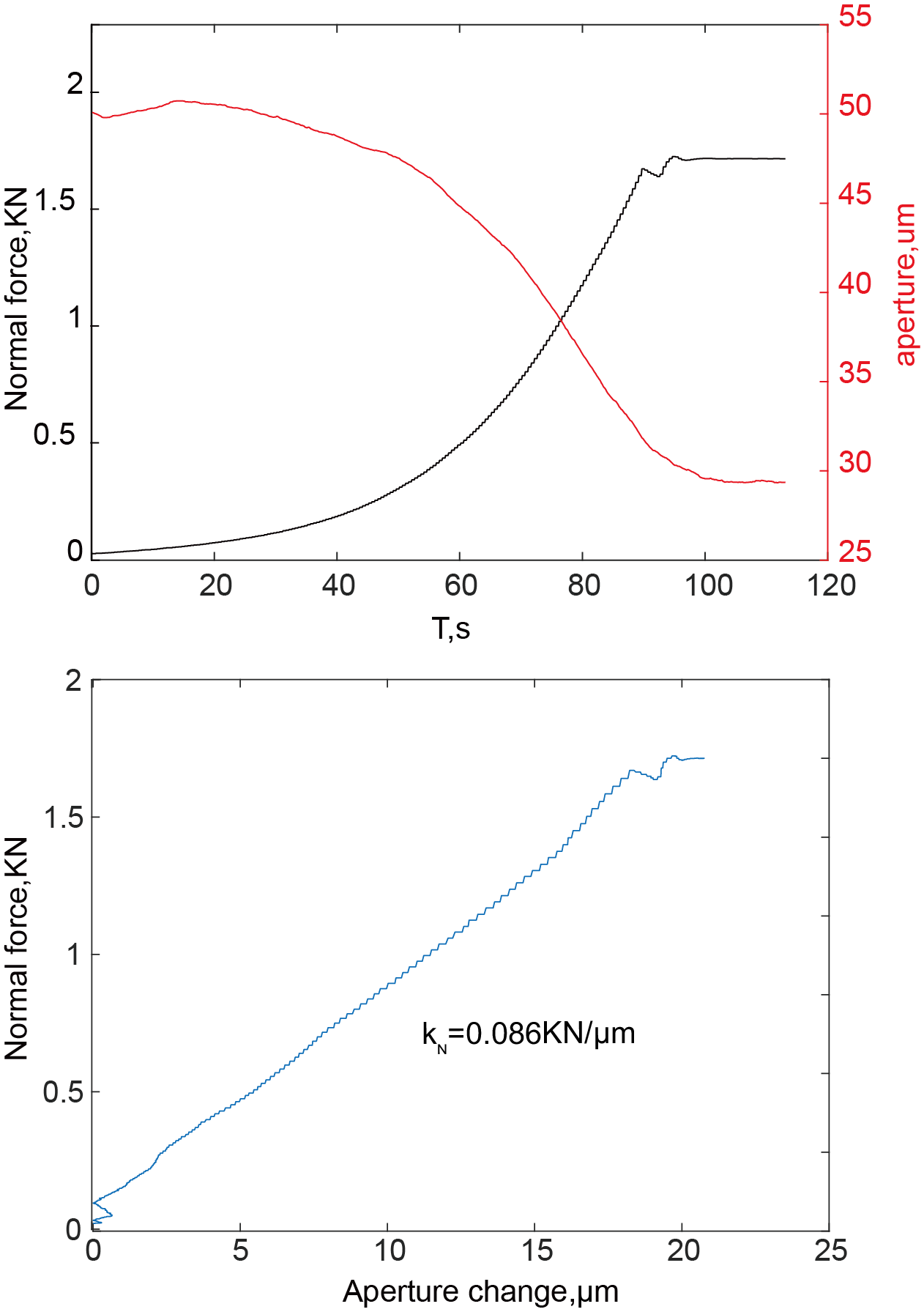


Supplementary Figure 3-The stiffness of fracture. a. Normal stress (black) and aperture change (red) with time during confining. b. Normal stress vs. aperture change with a slope that equal to stiffness of fractures.

Supplementary Notes

Normalizing seismic moment of laboratory and natural faults.

The maximum seismic moment induced by fluid pressure under laboratory conditions can be estimated based on the following assumptions:

(1). The stiffness of the experimental system is determined by the elastic interaction between the specimen and testing machine, and therefore is assumed to be constant.

(2). The laboratory fault is fully saturated before fluid injection.

(3). During the experiments, de-aired water is sequentially injected into the fully saturated fault at 0.1 MPa/min to elevate the fluid pressure, and this is sufficiently slow to allow fluid pressure to be uniform at each pressure step. We observe that fluid pressures reach equilibrium rapidly with injection, indicating that the laboratory fault is uniformly pressurized along its full length.

(4). Based on (2) and (3), the pressurized area and laboratory fault slip area are equivalent. Therefore, the induced ruptures are always confined within the pressurized zone. There is no situation where ruptures extend beyond the pressurized region in our experiments.

The total moment magnitude resulting from the reactivation of the laboratory fault is defined as $\sum M_{0}=AGu$ (1)

where $\sum M_{0}$ is the cumulative moment magnitude, $A$ is the area of the laboratory fault, $G$ is the modulus of rigidity, $u$ is the total shear slip displacement of laboratory fault, and this can be estimated by the shear stiffness of the experimental system$k_{s}$.

$\mathbf{k}_{\mathbf{s}}\mathbf{=}\frac{\mathbf{F}_{\mathbf{s}}}{\mathbf{u}}\mathbf{=}\frac{\mathbf{A}\boldsymbol{\Delta\tau}}{\mathbf{u}}$ (2)

where $\mathbf{F}_{\mathbf{s}}$ is the shear force that is provided by the axial load from pump B. $\boldsymbol{\Delta\tau}$ is the shear stress change resulting from the shear displacement.

According to the effective stress law (Biot coefficient of 1) with consideration of all possible initial stress states (c varies from 0 to 1), an increase in pore pressure ∆P is sufficient to cause fault failure and rupture as

$\mu\Delta P=\left( 1-c \right)\Delta\tau$ (3)

Combining equations (1), (2), and (3) gives the relation between seismic moment with increased pore pressure

$\mathbf{M=G}\frac{\boldsymbol{\mu}\mathbf{A}^{\mathbf{2}}}{\mathbf{(1-c)}\mathbf{k}_{\mathbf{s}}}\boldsymbol{\Delta P}$ (4)

Based on assumption (1), the total injected fluid volume is primarily accommodated by fault dilation, defined as

$\boldsymbol{\Delta V=}\boldsymbol{\Delta}\boldsymbol{a}\mathbf{A}$ (5)

where $\boldsymbol{\Delta a}$ is change in the aperture of the two fractures in the double-direct-shear sample core, and can be calculated by fracture normal stiffness ($\mathbf{k}_{\mathbf{n}}$) given by the change in pore pressure.

$\mathbf{k}_{\mathbf{n}}\mathbf{=}\frac{\boldsymbol{A\Delta}\boldsymbol{\sigma}_{\mathbf{N}}^{\mathbf{'}}}{\mathbf{u}_{\mathbf{n}}}\mathbf{=}\frac{\boldsymbol{A \Delta}\left( \boldsymbol{\sigma}_{\mathbf{N}}\mathbf{-}\mathbf{p}_{\mathbf{p}} \right)}{\boldsymbol{\Delta}\boldsymbol{a}}$ (6)

where $\boldsymbol{\sigma}_{\mathbf{N}}^{\mathbf{'}}$ is the effective normal stress and$\mathbf{u}_{\mathbf{n}}$ is normal displacement. As normal stress ($\boldsymbol{\sigma}_{\mathbf{N}}$) is controlled by the constant confining pressure, eqn (6) can be simplified as

$\mathbf{k}_{\mathbf{n}}\mathbf{=}\frac{\mathbf{A}\boldsymbol{\Delta P}}{\boldsymbol{\Delta}\boldsymbol{a}}$ (7)

The increased pore pressure can be related to the total injection volume by combing equations (5) and (7)

$\boldsymbol{\Delta V=}\frac{\mathbf{A}^{\mathbf{2}}\boldsymbol{\Delta P}}{\mathbf{k}_{\mathbf{n}}}$ (8)

If we substitute equation (8) into (4), we obtain a relationship between seismic moment vs total injection volume based on our laboratory conditions as (J.-P. Ampuero, Pers. Comm.),

$\boldsymbol{\sum M=}\frac{\boldsymbol{\mu}\mathbf{k}_{\mathbf{n}}}{\mathbf{k}_{\mathbf{s}}}\frac{\mathbf{1}}{\mathbf{(1-c)}}\boldsymbol{G\Delta V}$ (9)

If the earthquake magnitude-frequency relation follows the Gutenberg-Richter distribution, with coefficient of friction assumed as~0.6 and the b-value set as 1, the maximum anticipated moment magnitude from the laboratory observations ${\mathbf{M}_{\mathbf{0}}\mathbf{(lab)}}^{\mathbf{max}}$is equivalent to $\frac{\mathbf{1}}{\mathbf{2}}\boldsymbol{\sum M}$, with a detailed derivation found though eqn (7) to eqn(13) ^17^.

${\mathbf{M}_{\mathbf{0}}\mathbf{(lab)}}^{\mathbf{max}}\mathbf{=}\frac{\boldsymbol{\mu}\mathbf{k}_{\mathbf{n}}}{\mathbf{k}_{\mathbf{s}}}\frac{\mathbf{1}}{\mathbf{2(1-c)}}\boldsymbol{G\Delta V}$ (10)

where the rigid-body translations around the laboratory faults are linked to an equivalent modulus *via* fracture and system stiffnesses. Since the laboratory and natural faults are at different scales and under various constraints, the seismic moment estimated under laboratory conditions must be scaled to the seismic moment under field conditions. Comparing ${\mathbf{M}_{\mathbf{0}}\mathbf{(lab)}}^{\mathbf{max}}$ to the maximum moment magnitude induced by a natural fault in an homogeneously deforming elastic medium (i.e., McGarr, 2014) as

${\mathbf{M}_{\mathbf{0}}}^{\mathbf{max}}\mathbf{=}\frac{\mathbf{1}}{\mathbf{2(1-c)}}\boldsymbol{G\Delta V}$ (11)

requires the application of a scale factor defined as $\boldsymbol{\lambda=\mu}\frac{\mathbf{k}_{\mathbf{n}}}{\mathbf{k}_{\mathbf{s}}}$. Thus, the normalized maximum seismic moment for our laboratory slip events are defined as

${\mathbf{M'}_{\mathbf{0}}}^{\mathbf{max}}\mathbf{=}{\mathbf{M}_{\mathbf{0}}}^{\mathbf{max}}\boldsymbol{/\lambda}$ (12)

Supplementary Table

| Seismic Moments and Total Injected Volumes Sequence | | | | | | |
| --- | --- | --- | --- | --- | --- | --- |
| Event | ΔV(m3) | M0(max) | Type | Location | Reference | Reference |
| SJ | 6.50E+04 | 8.91E+15 | frack | Fort St. John ,Canada | Mahani et al. (2017) | 1 |
| POH | 1.28E+04 | 2.00E+17 | egs | Pohang (PX-2),South Korea | Grigoli et al. (2018),  Kim et al. (2018) | 2,3 |
| SG | 7.00E+02 | 1.00E+14 | egs | St. Gallen, Switzerland | Diehl et al. (2017) | 4 |
| GSF | 1.31E+00 | 6.21E+10 | scientific | southeast France | Guglielmi et al., ( 2015) | 5 |
|  | 2.80E+07 | 1.12E+15 | wd | Venus, Texas | Scales et al. (2017) | 6 |
|  | 1.45E+07 | 1.58E+15 | wd | Dagger Draw, New Mexico | Sanford et al. (2006) | 7 |
|  | 1.80E+06 | 8.91E+15 | wd | Tejon, Central Valley (WWF),  Kern County, California | Goebel et al. (2016) | 8 |
|  | 6.25E+05 | 2.11E+16 | wd | Rocky Mountain  Arsenal (Denver), Colorado | Healy et al. (1968) | 9 |
|  | 4.90E+06 | 1.00E+17 | wd | Raton Basin,  Colorado and New Mexico | Rubenstein et al. (2014) | 10 |
|  | 2.00E+02 | 5.62E+05 | frack | Western Canada | Maxwell et al. (2009) | 11 |
|  | 1.10E+03 | 5.62E+08 | frack | Cotton Valley, Texas | Urbancic et al. (1999) | 12 |
|  | 9.42E+04 | 2.24E+12 | frack | Ryser well,  Harrison County, Ohio | Friberg et al. (2014) | 13 |
|  | 3.02E+05 | 1.26E+13 | frack | Fox Creek (Well Pad 4) | Bao and Eaton (2016) | 14 |
|  | 1.26E+05 | 2.51E+13 | frack | Fox Creek (Well Pad 2) | Bao and Eaton (2016) | 14 |
|  | 4.03E+04 | 2.51E+13 | frack | Fox Creek (Well Pad 5) | Bao and Eaton (2016) | 14 |
|  | 3.74E+04 | 3.55E+13 | frack | Fox Creek (Well Pad 6) | Bao and Eaton (2016) | 14 |
|  | 2.68E+04 | 3.98E+14 | frack | Fox Creek (Well Pad 3) | Bao and Eaton (2016) | 14 |
|  | 6.11E+04 | 7.94E+14 | frack | Fox Creek (Well Pad 1) | Bao and Eaton (2016) | 14 |
|  | 1.30E+04 | 3.55E+07 | egs | Groẞ-Schönebeck,Germany | Zang et al. (2014) | 15 |
|  | 5.60E+03 | 5.62E+11 | egs | Bad Urach,Germany | Evans et al. (2012) | 16 |
|  | 1.01E+04 | 1.12E+12 | egs | Ogachi (OGC-1),Japan | Kaieda et al. (2010) | 17 |
|  | 4.13E+04 | 4.32E+12 | egs | Newberry,US | Breede et al. (2013) | 18 |
|  | 3.10E+03 | 6.31E+12 | egs | Well Paralana 2,Australia | Albaric et al. (2014) | 19 |

Reference

1. Mahani, A. B. et al. Fluid injection and seismic activity in the Northern Montney Play, British Columbia, Canada, with special reference to the 17 August 2015 Mw 4.6 induced earthquake. Bull. Seismol. Soc. Am. 107, 542–552 (2017).
2. Grigoli, F. et al. The November 2017 M w 5.5 Pohang earthquake: a possible case of induced seismicity in South Korea. Science 360, 1003–1006 (2018).
3. Kim, K. H. et al. Assessing whether the 2017 Mw5.4 Pohang earthquake in South Korea was an induced event. Science 360, 1007–1009 (2018).
4. Diehl, T., Kraft, T., Kissling, E. & Wiemer, S. The induced earthquake sequence related to the St. Gallen deep geothermal project (Switzerland): fault reactivation and fluid interactions imaged by microseismicity. J. Geophys. Res. Solid Earth 122, 7272–7290 (2017).
5. Guglielmi, Y., Cappa, F., Avouac, J. P., Henry, P. & Elsworth, D. Seismicity triggered by fluid injection-induced aseismic slip. Science 348, 1224–1226 (2015).
6. Scales, M. M. et al. A Decade of Induced Slip on the Causative Fault of the 2015 Mw 4.0 Venus Earthquake, Northeast Johnson County, Texas. J. Geophys. Res. Solid Earth 122, 7879–7894 (2017).
7. Sanford, A. R., Mayeau, T. M., Schlue, J. W., Aster, R. C. & Jaksha, L. H. Earthquake catalogs for New Mexico and bordering areas II: 1999-2004. N. Mex. Geol. 28, 99–109 (2006).
8. Goebel, T. H. W. et al. Wastewater disposal and earthquake swarm activity at the southern end of the Central Valley, California. Geophys. Res. Lett. 43,1092–1099 (2016).
9. Healy, J. H., Rubey, W. W., Griggs, D. T. & Raleigh, C. B. The Denver Earthquakes. Disposal of waste fluids by injection into a deep well has riggered earthquakes near Denver, Colorado. Science 161, 1301–1310 (1968).
10. Rubinstein, J. L., Ellsworth, W. L., McGarr, A. & Benz, H. M. The 2001–present induced earthquake sequence in the Raton Basin of northern New Mexico and southern Colorado. Bull. Seismol. Soc. Am. 104, 2162–2181 (2014).
11. Maxwell, S. C., Shemeta, J., Campbell, E. & Quirk, D. Microseismic deformation rate monitoring. Proc. - SPE Annu. Tech. Conf. Exhib. 6, 4185–4193 (2008).
12. Urbancic, T. I., Shumila, V., Rutledge, J. T. & Zinno, R. J. Determining hydraulic fracture behavior using microseismicity. Vail Rocks 1999 - 37th U.S. Symp. Rock Mech. 991–997 (1999).
13. Friberg, P. A., Besana-Ostman, G. M. & Dricker, I. Characterization of an earthquake sequence triggered by hydraulic fracturing in Harrison county, Ohio. Seismol. Res. Lett. 85, 1295–1307 (2014).
14. Bao, X. & Eaton, D. W. Fault activation by hydraulic fracturing in western Canada. Science 354, 1406–1409 (2016).
15. Zang, A. et al. Analysis of induced seismicity in geothermal reservoirs – An overview. Geothermics 52, 6–21 (2014).
16. Evans, K. F., Zappone, A., Kraft, T., Deichmann, N. & Moia, F. A survey of the induced seismic responses to fluid injection in geothermal and CO_2_ reservoirs in Europe. Geothermics 41, 30–54 (2012).
17. Kaieda, H., Sasaki, S. & Wyborn, D. Comparison of Characteristics of Micro-Earthquakes Observed During Hydraulic Stimulation Operations in Ogachi. Hijiori Cooper Basin HDR Proj. World Geotherm. Congr. 2010, 1–6 (2010).
18. Breede, K., Dzebisashvili, K., Liu, X. & Falcone, G. A systematic review of enhanced (or engineered) geothermal systems: past, present and future. Geotherm. Energy 1, 4 (2013).
19. Albaric, J. et al. Monitoring of induced seismicity during the first geothermal reservoir stimulation at Paralana, Australia. Geothermics 52, 120–131 (2014).
